# Supplementary material for: Some performance considerations when using multi-armed bandit algorithms in the presence of missing data
Source: PLoS One. 2022 Sep 12;17(9):e0274272. doi: 10.1371/journal.pone.0274272 (PMC9467360; doi:10.1371/journal.pone.0274272)
Supplement: S1 Appendix — (PDF) [file pone.0274272.s001.pdf]

Algorithm 1: Implementation of bandit algorithms with missing data

Input: trial size n; probability of success p_k; probability of missingness p_k^{m}.

Initialization: The initial prior $x_{k,0} = (s_{k,0},\ f_{k,0})$. **while** $t < n$ **do**

    Decision for allocation based on the current state $x_{k,t} = (s_{k,0} + S_{k,t},\ f_{k,0} + F_{k,t})$;
    if *observed* $(p_k^m < u,\ u \sim U_{[0,1]})$ **then**
        $M_{k,t}$ += 0.
        if *failure* $(p_k < u,\ u \sim U_{[0,1]})$ **then**
            $F_{k,t}$ += 1;
            $S_{k,t}$ += 0;
        else
            $S_{k,t}$ += 1;
            $F_{k,t}$ += 0;
        end
    else
        $M_{k,t}$ += 1; $F_{k,t}$ += 0; $S_{k,t}$ += 0;
    end
    Update $x_{k,t}$ with $(S_{k,t},\ F_{k,t})$ for the next allocation

end

Output: $(S_{k,n},\ F_{k,n},\ M_{k,n})$
