# Supplementary material for: Some performance considerations when using multi-armed bandit algorithms in the presence of missing data
Source: PLoS One. 2022 Sep 12;17(9):e0274272. doi: 10.1371/journal.pone.0274272 (PMC9467360; doi:10.1371/journal.pone.0274272)
Supplement: S2 Appendix — (PDF) [file pone.0274272.s002.pdf]

Algorithm 2: Implementation of bandit algorithms with missing data and imputation

Input: trial size n; probabilities of success p_k; probabilities of missingness p_k^{m}.
Initialization: The initial prior $x_{k,0} = (s_{k,0},\ f_{k,0})$.
while $t < n$ **do**
  Decision for allocation based on the current state $x_{k,t} = (s_{k,0} + S_{k,t},\ f_{k,0} + F_{k,t})$;
  if *observed* ($p_k^m < u,\ u \sim U_{[0,1]}$) **then**
    if *failure* ($p_k < u,\ u \sim U_{[0,1]}$) **then**
      $F_{k,t}$ += 1;
      $S_{k,t}$ += 0;
    else
      $S_{k,t}$ += 1;
      $F_{k,t}$ += 0;
    end
  else
    Imputation based on $\hat{p}_{k,t}$;
    if *failed* ($\hat{p}_{k,t} < u,\ u \sim U_{[0,1]}$) **then**
      $F_{k,t}^{\mathrm{imp}}$ += 1;
      $S_{k,t}^{\mathrm{imp}}$ += 0;
    else
      $S_{k,t}^{\mathrm{imp}}$ += 1;
      $F_{k,t}^{\mathrm{imp}}$ += 0;
    end
  end
  Update $x_{k,t}$ with $(S_{k,t} + S_{k,t}^{\mathrm{imp}},\ F_{k,t} + F_{k,t}^{\mathrm{imp}})$ for the next allocation
end
Output: $(S_{k,n} + S_{k,n}^{\mathrm{imp}},\ F_{k,n} + F_{k,n}^{\mathrm{imp}})$
