# Supplementary figures and images for: Some performance considerations when using multi-armed bandit algorithms in the presence of missing data
Source: PLoS One. 2022 Sep 12;17(9):e0274272. doi: 10.1371/journal.pone.0274272 (PMC9467360; doi:10.1371/journal.pone.0274272)

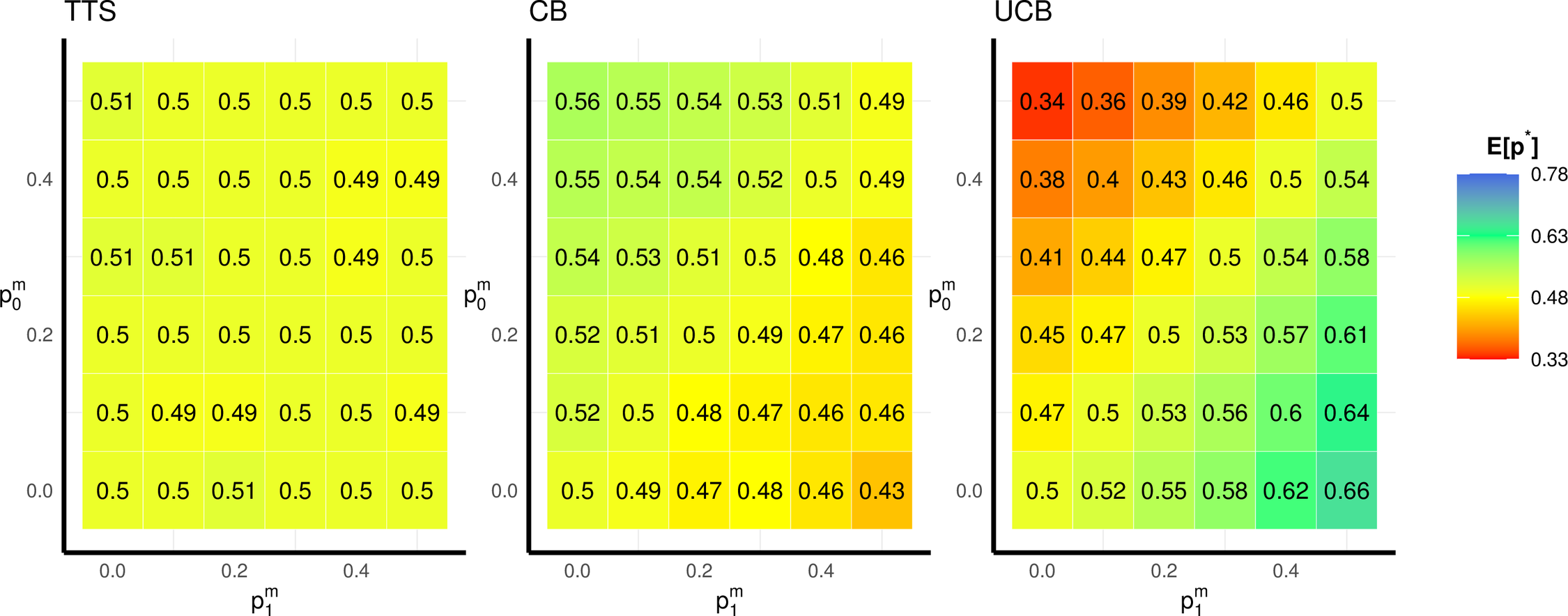

Supplement: S3 Appendix — Expectations of 104 replications are taken for CB and UCB and 103 replications for TTS under different combinations of missingness probabilities, with p0 = p1 = 0.7 and n = 200. (TIF) [file pone.0274272.s003.tif]

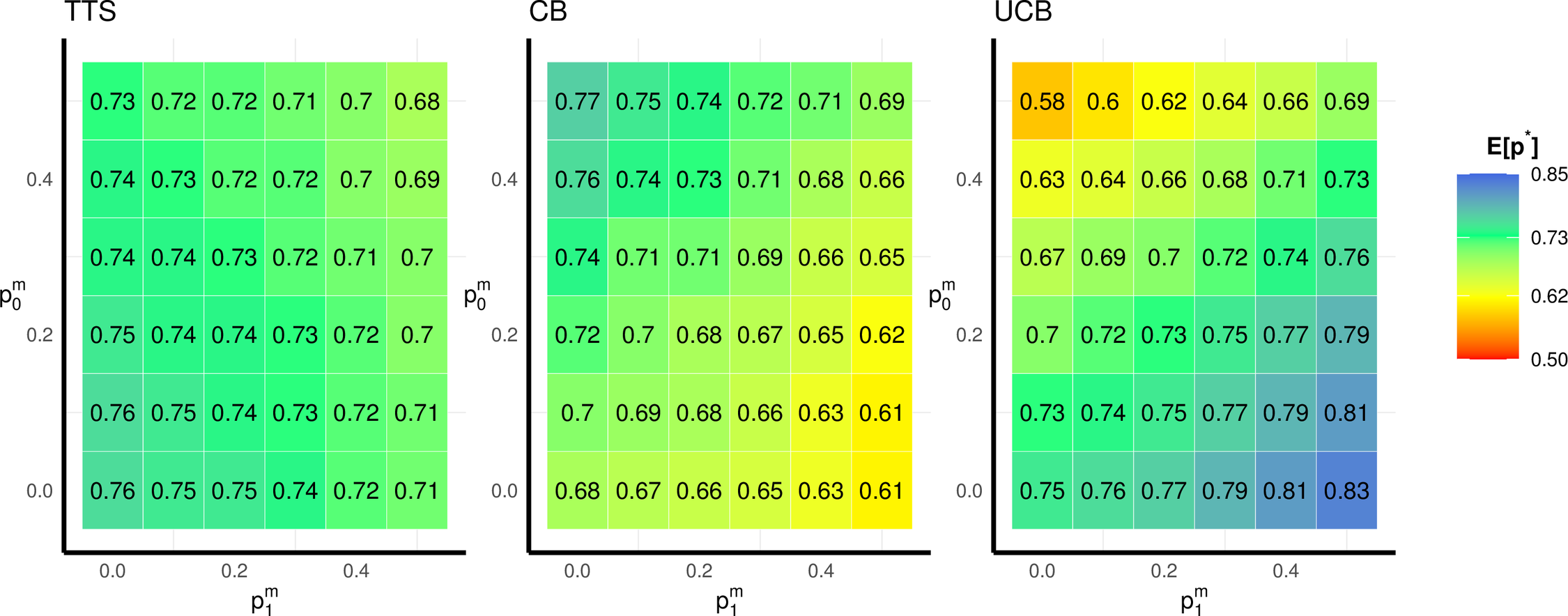

Supplement: S4 Appendix — Expectations of 104 replications are taken for CB and UCB and 103 replications for TTS under different combinations of missingness probabilities, with p0 = 0.7, p1 = 0.9, and n = 200. (TIF) [file pone.0274272.s004.tif]

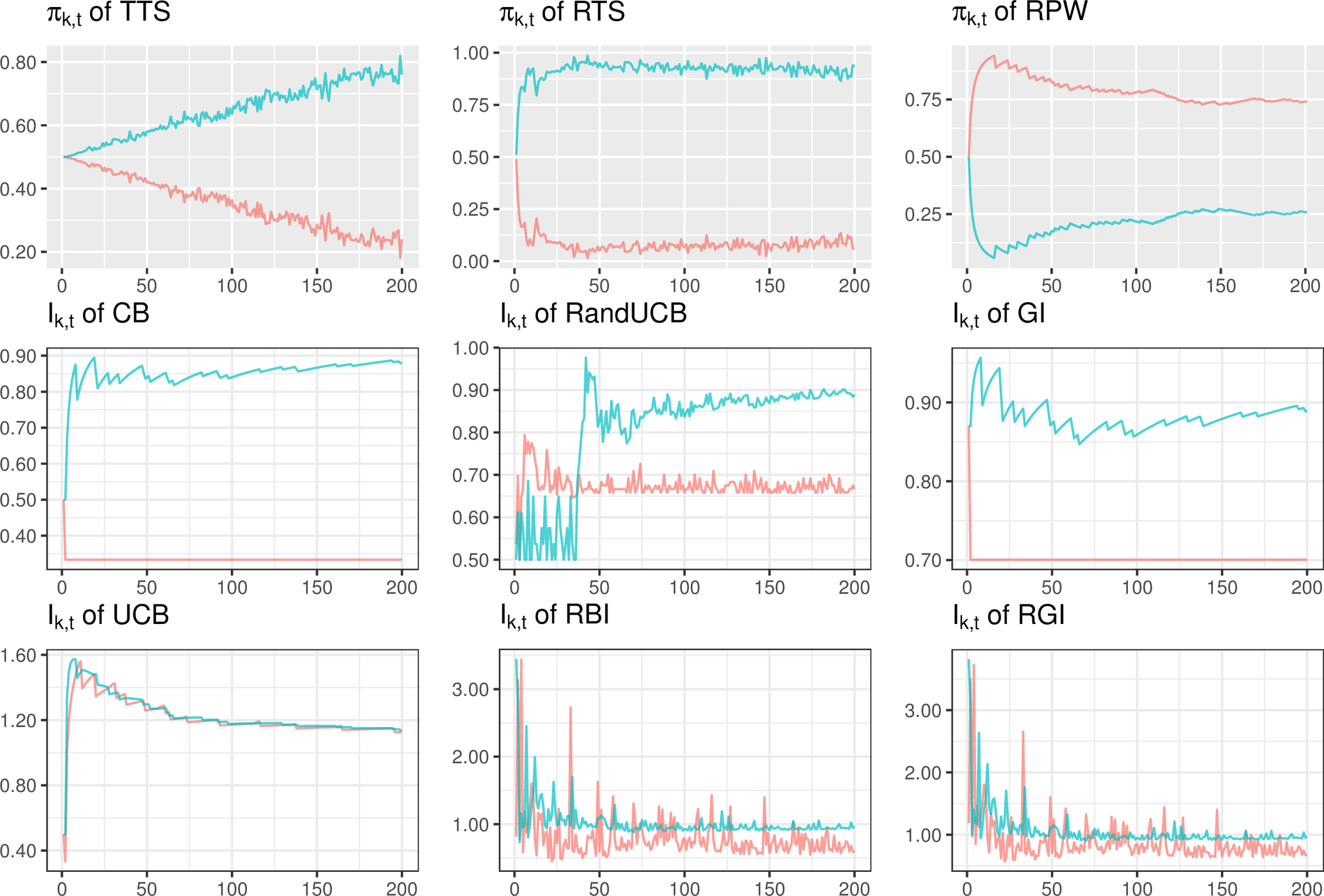

Supplement: S5 Appendix — Blue line represent πk,t or Ik,t in the experimental arm and red lines represent that in the control arm. (TIF) [file pone.0274272.s005.tif]

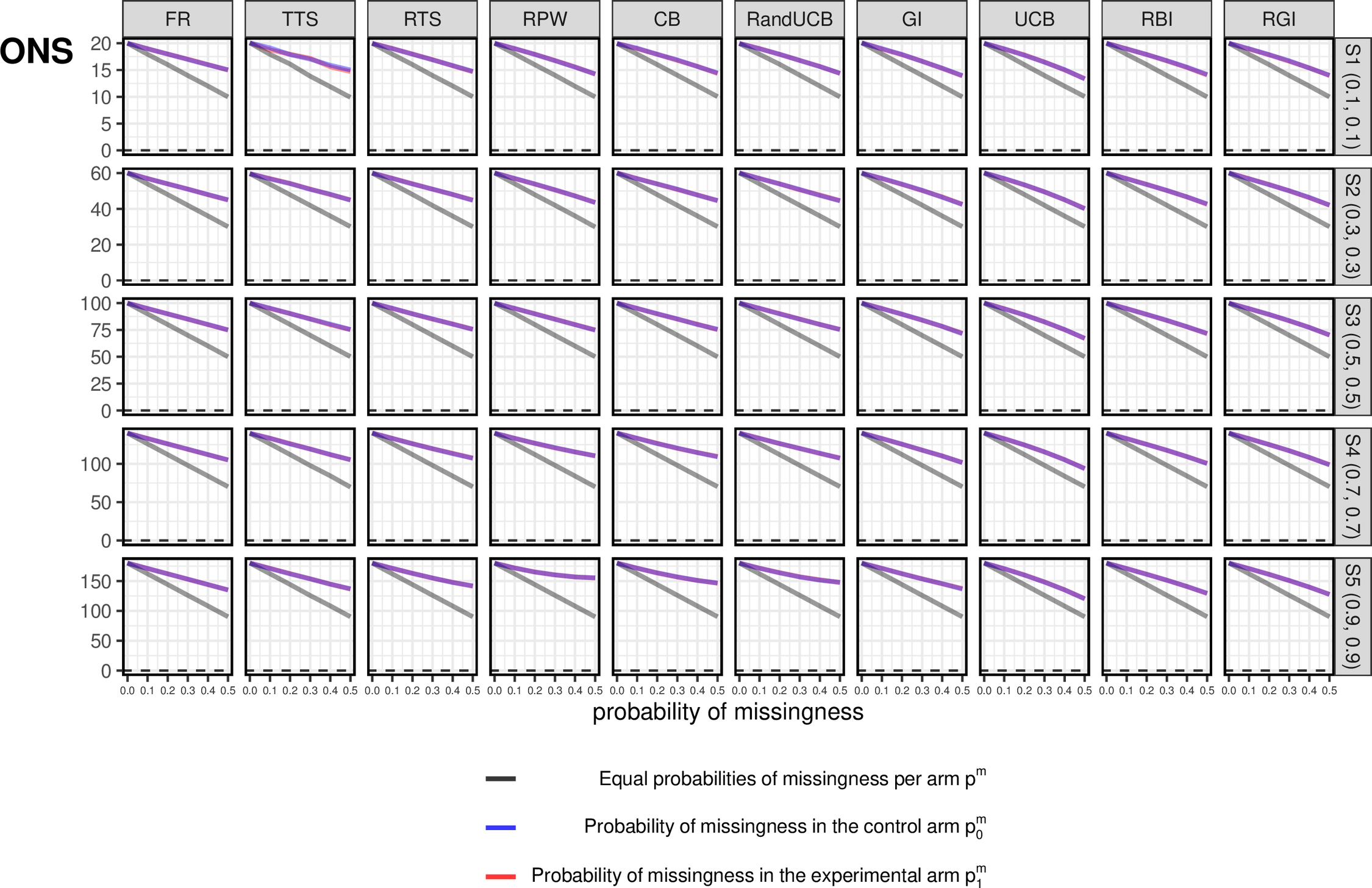

Supplement: S6 Appendix — The impact of missing data is similar under different algorithms under the null: the impact due to the equal probabilities of missing data per arm is larger than the impact of only having missingness in the control or experimental arm. (TIF) [file pone.0274272.s006.tif]

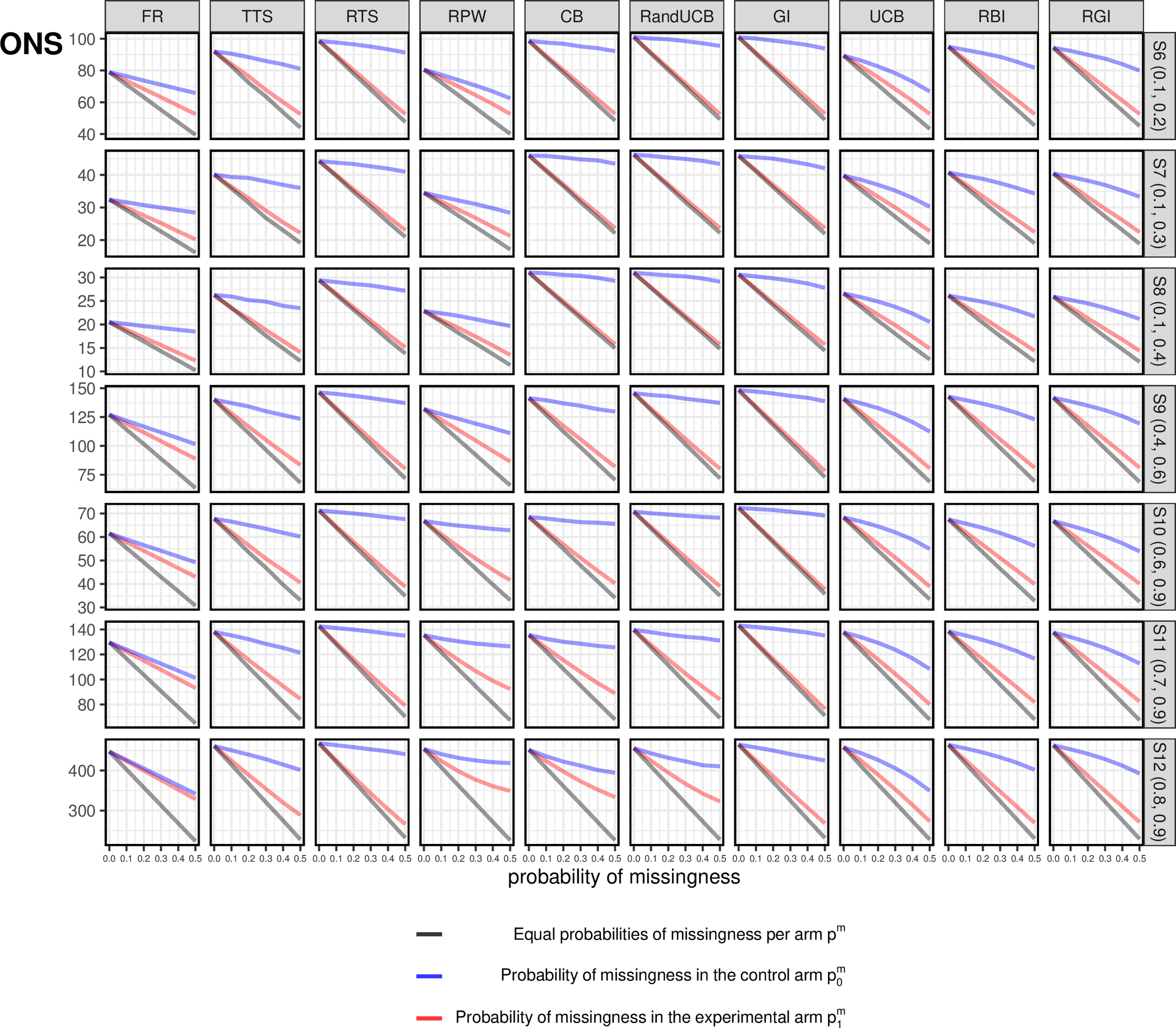

Supplement: S7 Appendix — The impact of missing data on ONS varies among different algorithms. Generally, bandit algorithms outperform FR when there is no missing data. Equal missingness is a more serious problem than missingness only occurring in the experimental arm, which in turn is a much more serious problem than missingness only occurring in the control arm. (TIF) [file pone.0274272.s007.tif]

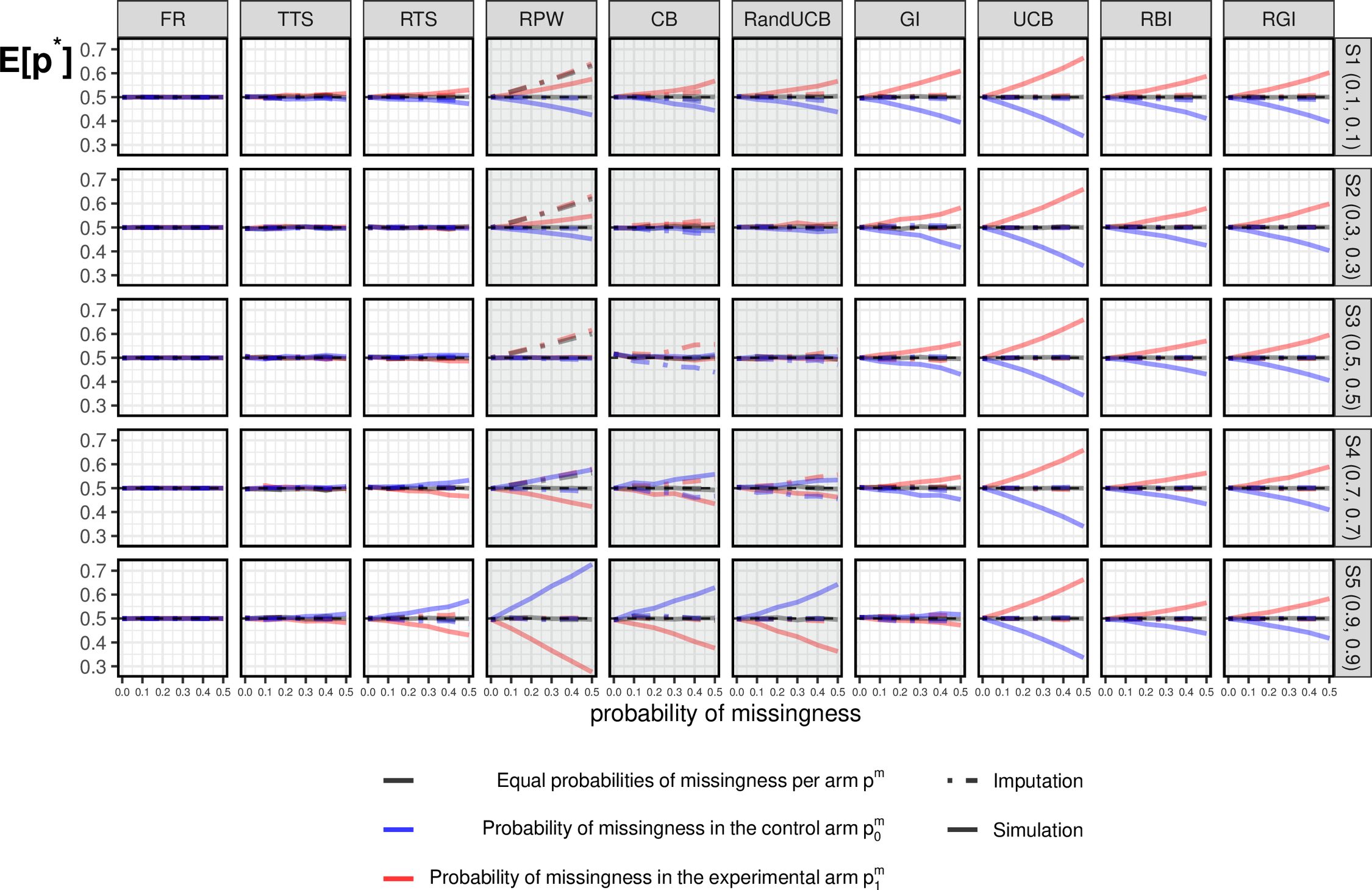

Supplement: S8 Appendix — Imputation results of E[p*] under the null for different missing data combinations with initial value p^k,0=0.9. Grey lines correspond to the case of equal missingness probability in both arms; Blue lines correspond to missingness in the control arm; Red lines correspond to missingness in the experimental arm. Solid lines correspond to the results without mean imputation, while the dashed lines correspond to the results with mean imputation. (TIF) [file pone.0274272.s008.tif]

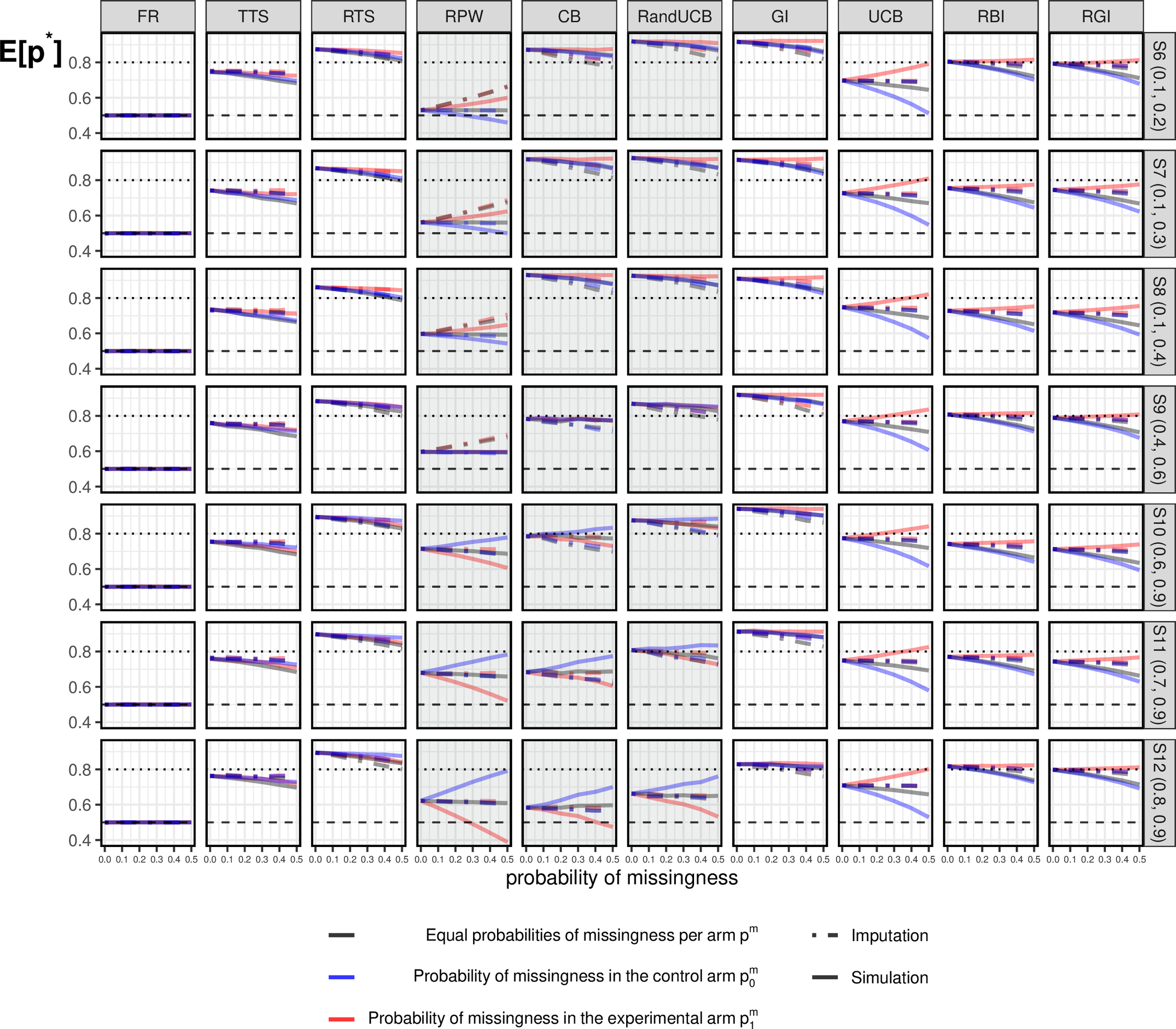

Supplement: S9 Appendix — Imputation results of E[p*] under the alternative for different missing data combinations with initial value p^k,0=0.9. Grey lines correspond to the case of equal missingness probability in both arms; Blue lines correspond to missingness in the control arm; Red lines correspond to missingness in the experimental arm. Solid lines correspond to the results without mean imputation, while the dashed lines correspond to the results with mean imputation. (TIF) [file pone.0274272.s009.tif]

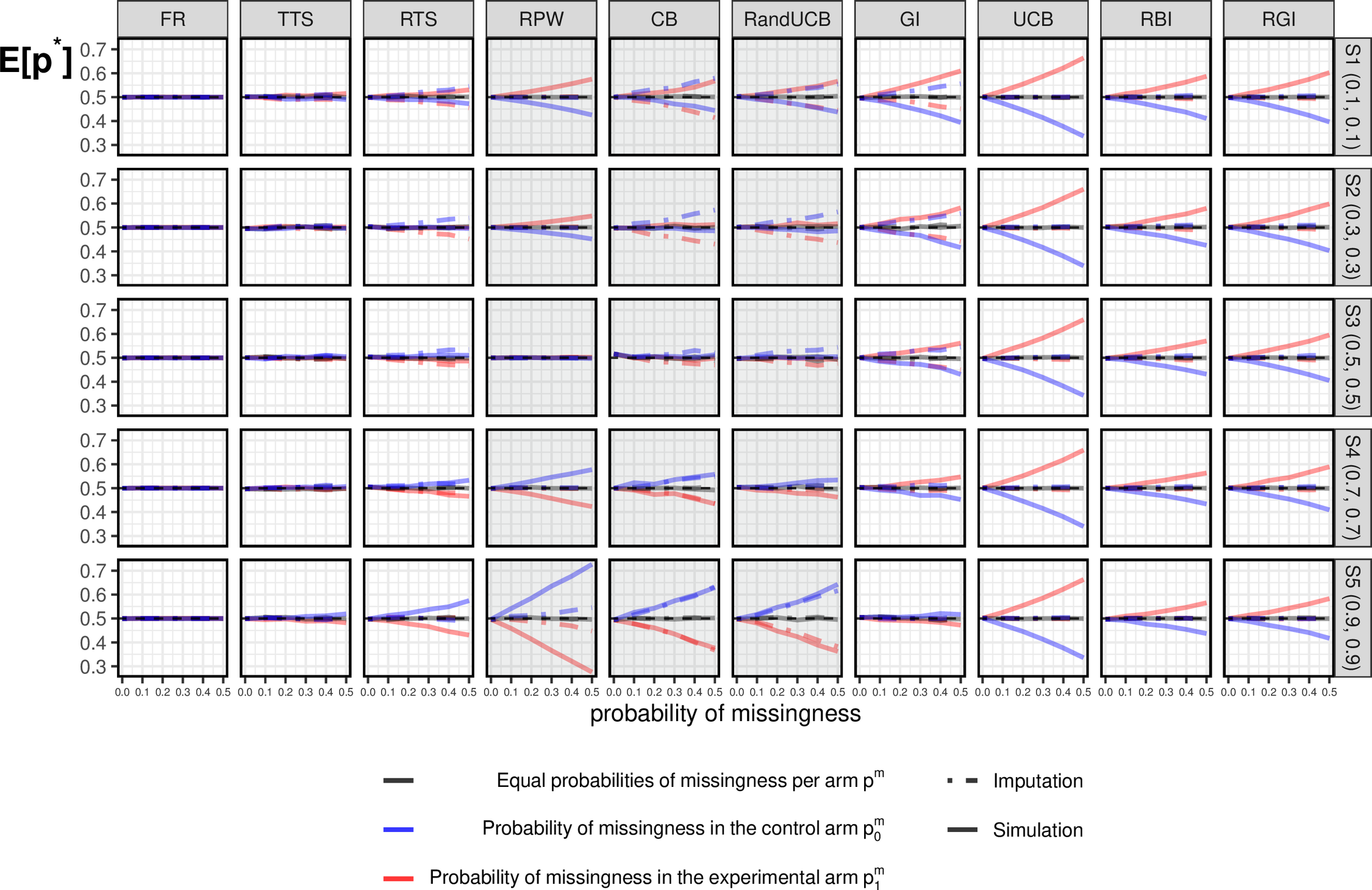

Supplement: S10 Appendix — Imputation results of E[p*] under the null for different missing data combinations, with imputation starting after the first observation. Grey lines correspond to the case of equal missingness probability in both arms; Blue lines correspond to missingness in the control arm; Red lines correspond to missingness in the experimental arm. Solid lines correspond to the results without mean imputation, while the dashed lines correspond to the results with mean imputation. (TIF) [file pone.0274272.s010.tif]

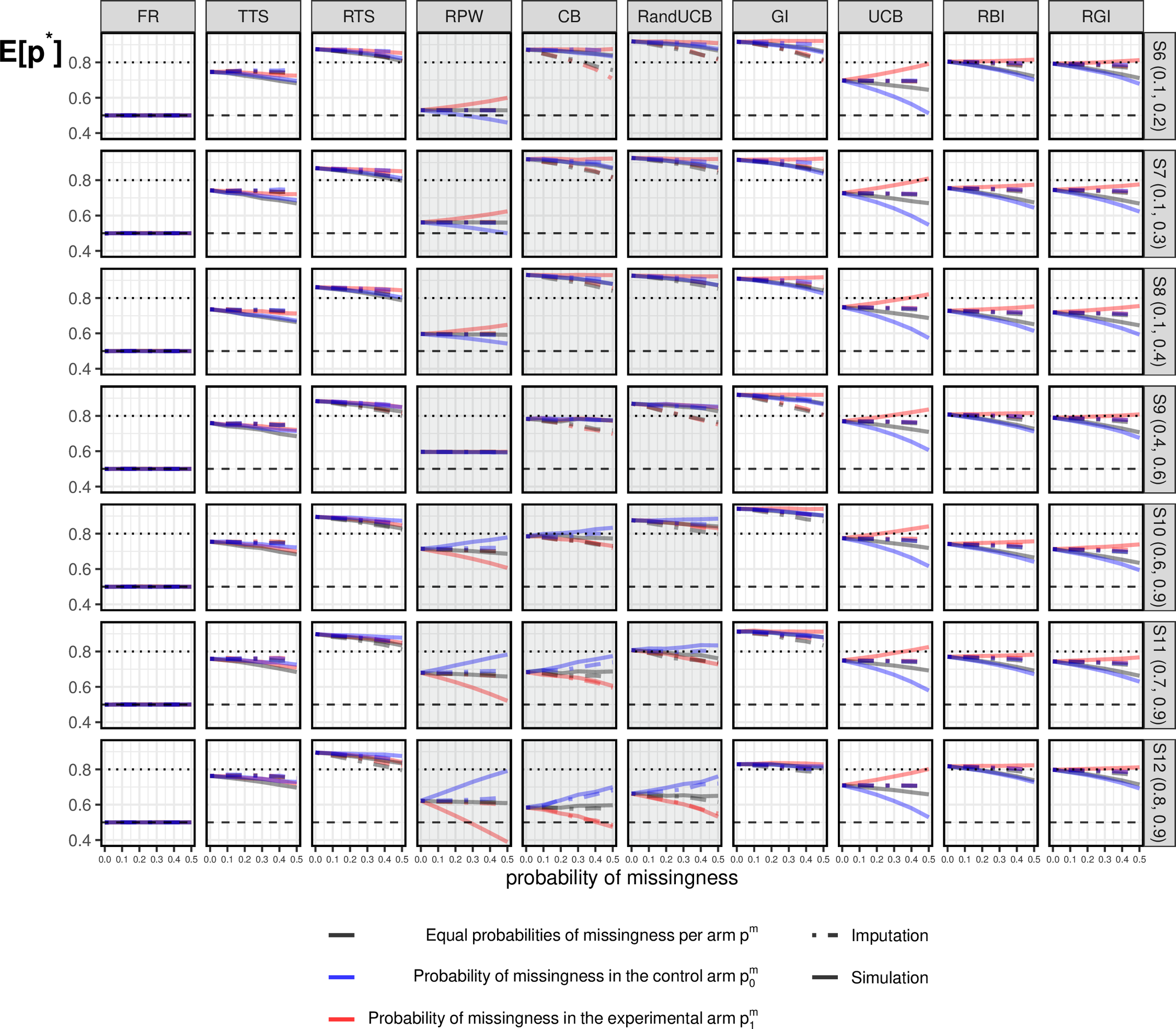

Supplement: S11 Appendix — Imputation results of E[p*] under the alternative for different missing data combinations, with imputation starting after the first observation. Grey lines correspond to the case of equal missingness probability in both arms; Blue lines correspond to missingness in the control arm; Red lines correspond to missingness in the experimental arm. Solid lines correspond to the results without mean imputation, while the dashed lines correspond to the results with mean imputation. (TIF) [file pone.0274272.s011.tif]

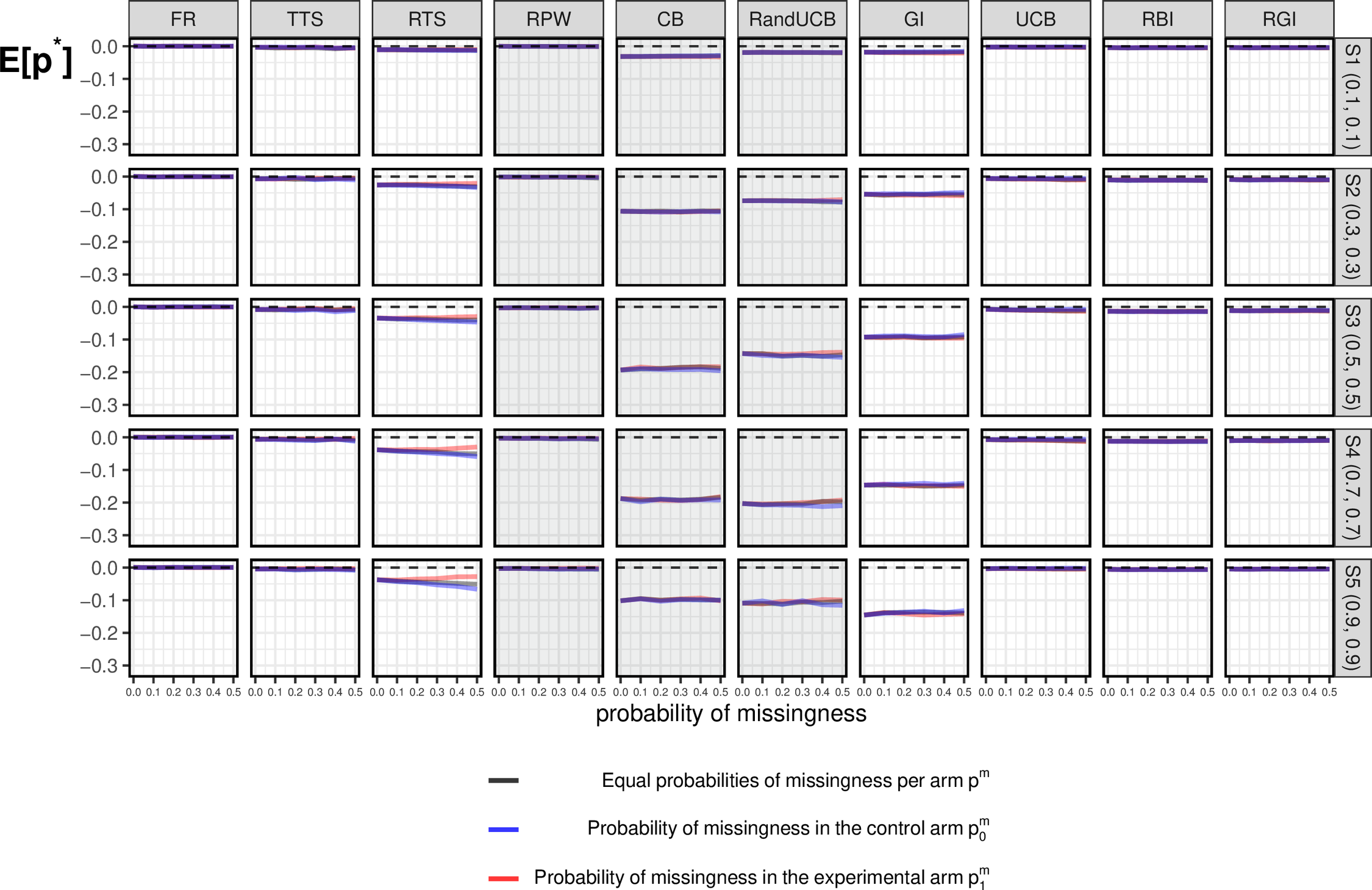

Supplement: S12 Appendix — Simulation results of p^k,t-pk,t under the null for different missing data combinations. We illustrate the result for one of the two equal arms. Grey lines correspond to the case of equal missingness probability in both arms; Blue lines correspond to missingness in the control arm; Red lines correspond to missingness in the experimental arm. (TIF) [file pone.0274272.s012.tif]

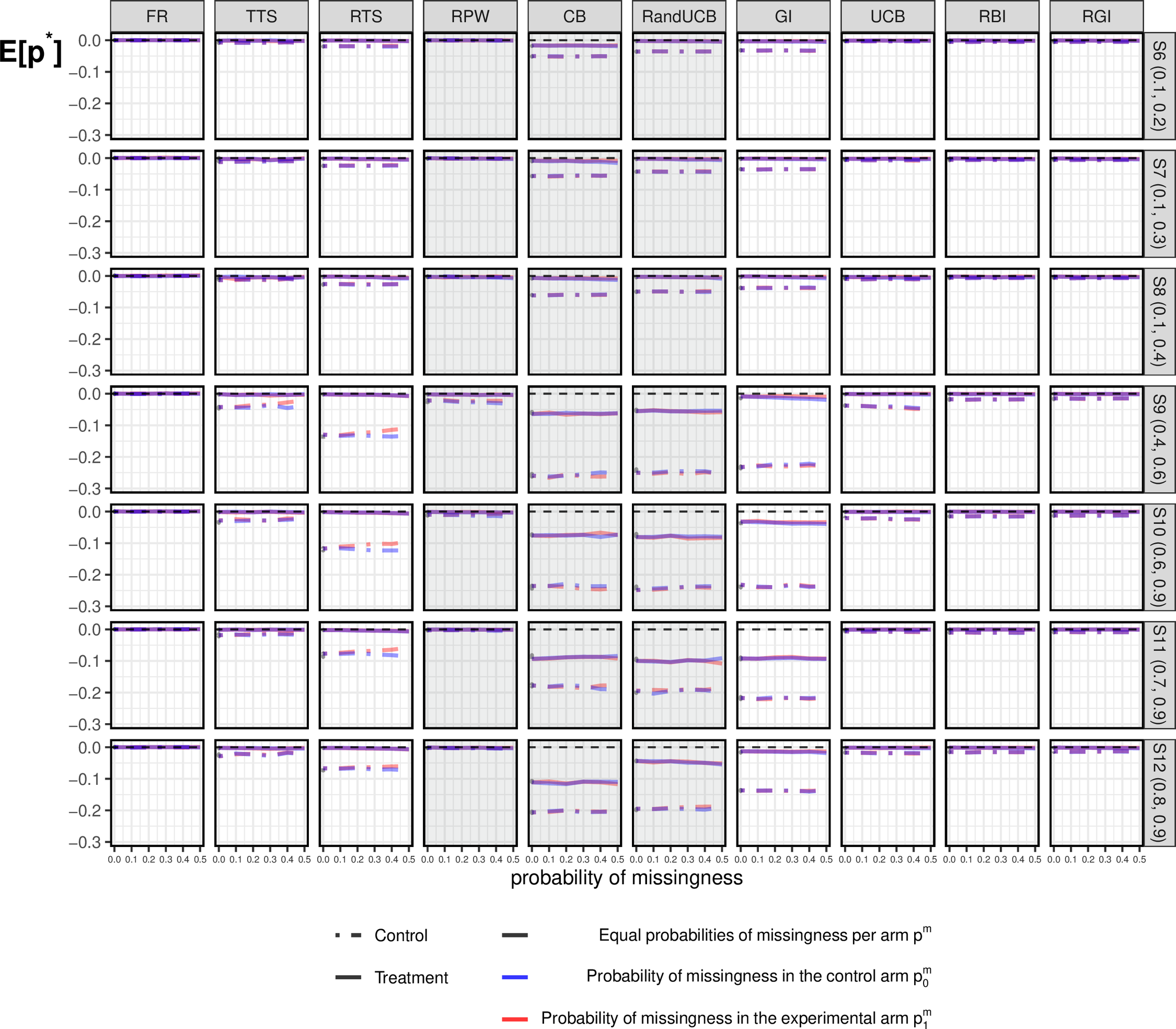

Supplement: S13 Appendix — Simulation results of p^k,t-pk,t under the alternative for different missing data combinations. We illustrate the result for one of the two equal arms. Grey lines correspond to the case of equal missingness probability in both arms; Blue lines correspond to missingness in the control arm; Red lines correspond to missingness in the experimental arm. Solid lines correspond to the bias in the treatment arm, while the dashed lines correspond to the bias in the control arm. (TIF) [file pone.0274272.s013.tif]
