# Supplementary material for: Some performance considerations when using multi-armed bandit algorithms in the presence of missing data
Source: PLoS One. 2022 Sep 12;17(9):e0274272. doi: 10.1371/journal.pone.0274272 (PMC9467360; doi:10.1371/journal.pone.0274272)
Supplement: Attachment — Submitted filename: Response.pdf [file pone.0274272.s014.pdf]

Some performance considerations when using multi-armed bandit algorithms in the presence of missing data

Response to Reviewers

Xijin Chen, Kim May Lee, Sofía S. Villar, David S. Roberston

Manuscript ID: PONE-D-22-10081

Dear Professor Worthy,

We would like to thank you, and the referee for the time and effort that have gone into the review. We very much appreciate the invitation to revise our manuscript for further consideration by PLOS One. We believe that the manuscript has been substantially improved as a result of incorporating the constructive and insightful suggestions received as part of this review process.

Please find below a report which provides a detailed, point-by-point response (in *italics*) to all the comments raised in the review. To help identify the changes made to the original manuscript, we have also provided a supplementary file (see 'Revised Manuscript with Track Changes') which highlights all changes we made in magenta color.

In addition to the specific comments raised, we have carefully checked the manuscript for typographical and grammatical errors.

I confirm that all authors have reviewed and approved the revision of this manuscript, and hence agree to its resubmission.

The funders had no role in study design, data collection and analysis, decision to publish, or preparation of the manuscript.

Thank you for considering the revised version of this manuscript which we hope will be suitable for publication in PLOS One. We look forward to hearing from you.

Yours sincerely,

Xjin Chen (on behalf of all authors)

Response to Reviewer

Review of existing literature: I think the following articles (and appropriate references therein) should be cited since they are related to the theme of this paper:

1. Why adaptively collected data have negative bias and how to correct for it [Nie et al., AISTATS 2018]

2. Are sample means in multi-armed bandits positively or negatively biased? [Shin et al., NeurIPS 2019]

3. Accurate inference for adaptive linear model [Deshpande et al. ICML 2018]

4. Online multi-armed bandits with adaptive inference [Dimakopoulou et al., NeurIPS 2021]

Aforementioned works investigate theoretically the directions, causes, implications and mitigation for biases in bandit algorithms resulting from sample-adaptivity, and are highly relevant to this paper, especially in the context of mean imputation for missing data. Cited references may be able to provide a theoretical explanation for many of the empirical observations made in this submission. In addition, reference [56] ("A closer look at the worst-case behavior of multi-armed bandit algorithms") provides a theoretical explanation for the 'imbalanced' behavior of RTS under the null, observable also in experimental results in this submission. The same reference also provides an explanation for the behavior of UCB1 (Auer et al. 2002) under the null; these aspects should be elucidated in detail in view of the numerical experiments conducted in this submission.

We thank the reviewer for pointing out the above theoretical references. We agree that including these are a very helpful addition and highly relevant to our paper, in particular in explaining the results seen when using mean imputation for missing data. We have now incorporated all of the reference into our paper in a new section 4.3 ('The impact of biased estimates on mean imputation'), as summarised below. This section is the major revision we have incorporated and is a direct response to the reviewer's insightful feedback that allowed us to better explain our results. Note also we have updated our abstract in an attempt to better present our main observations in light of our reading of the references suggested by the reviewer.

1. Nie et al. (2018) prove that the bias of the sample mean for any fixed arm and at any fixed time is negative when the sampling strategy satisfies two conditions called 'Exploit' and 'Independence of Irrelevant Options' (IIO). Besides, they suggest two ways targeting the biased estimate via modifying the data collection procedure. We discuss the mean imputation results with the explanations of negative bias in our revised manuscript and we mention this paper in Line 674, 676, 743 of the revised manuscript.

2. Shin et al. (2019) theoretically discuss that in many typical MAB settings, we should expect sample means to have two contradictory sources of bias: negative bias from 'optimistic sampling' and positive bias from 'optimistic stopping/choosing'. This not only provides broader discussion than the contexts in Nie et al. (2017) and our submission, which could be some directions for future research, but also this work extends the formula for negative bias given in Bowden and Trippa (2017) that only applied to randomised data adaptive sampling rules. The formula in both references give us some insights on the magnitude of bias in different multi-armed algorithms. We discuss this in combination with our experimental results to provide additional and new interpretations in Line 674, 678, 691 of the revised manuscript.

3. Deshpande et al. (2018) discuss the simple case of multi-armed bandits without covariates, where the ordinary least squares estimates correspond to computing sample means for each arm. They propose to decorrelate the OLS estimator. Even though this is not implemented to do imputation in our submission, we discuss how this could be an avenue for future investigation for the imputation of the missing data in Line 745 of the revised manuscript.

4. Dimakopoulou et al. (2021) proposed the Doubly-Adaptive Thompson Sampling (DATS) by harnessing the strengths of adaptive inference estimators to ensure sufficient exploration in the initial stages of learning and the effective exploration-exploitation balance provided by the TS mechanism. This debiasing technique is not used in our submission, but we discuss how this could be a way of handling the problem of biased estimate for some specific algorithms (i.e., TS) in Line 748 of the revised manuscript.

We also thank the reviewer for highlighting Kalvit and Zeevi (2021) (Reference [56]), which discusses the sampling behaviour of TS and UCB under the 'large gap' (i.e., 'well-separated') and 'small gap' (i.e., 'worst-case') instances. The latter setting matches the scenarios under the null in our experimental investigations. For this reason, this reference helps to explain the 'incomplete sampling' (i.e., 'random selection' in our submission) of TS from a theoretical perspective. This behaviour is different from the 'complete learning' behaviour of UCB (i.e. inducing a 'balanced' allocation under the null), which has also been seen in our experimental results. We have modified the related discussions to include this reference as an explanation of the sampling behaviour of TS and UCB in Line 300, 318, 335 of the revised manuscript.

Miscellaneous: Line 243 – Shouldn't it be 't' instead of 'T' in the expression of β?

We thank the reviewer for pointing out this typo, we have replaced T with t.
